# Korea National Health and Nutrition Examination Survey, 20th anniversary: accomplishments and future directions

**DOI:** 10.4178/epih.e2021025

**Published:** 2021-04-19

**Authors:** Kyungwon Oh, Yoonjung Kim, Sanghui Kweon, Soyeon Kim, Sungha Yun, Suyeon Park, Yeon-Kyeng Lee, Youngtaek Kim, Ok Park, Eun Kyeong Jeong

**Affiliations:** 1 Division of Health and Nutrition Survey and Analysis, Bureau of Chronic Disease Prevention and Control, Korea Disease Control and Prevention Agency, Cheongju, Korea; 2 Division of Healthcare Association Infection Control, Bureau of Healthcare Safety and Immunization, Korea Disease Control and Prevention Agency, Cheongju, Korea; 3 Public Health Medical Service Office, Chungnam National University Hospital, Daejeon, Korea; 4 Korea Disease Control and Prevention Agency, Cheongju, Korea

**Keywords:** Korea National Health and Nutrition Examination Survey, Health behaviors, Nutrition assessment, Chronic disease indicators

## Abstract

The Korea National Health and Nutrition Examination Survey (KNHANES) was initiated in 1998 to provide evidence for the development and evaluation of health policies and programs. The Korea Disease Control and Prevention Agency is responsible for the KNHANES and has conducted it as a series of surveys. Over the past 20 years, efforts to produce accurate, timely, and nationwide health statistics have been refined by establishing a continuous annual survey system with full-time field staff, incrementally expanding survey components, collaborating with relevant academic societies for quality control, and revising the survey methods. Additionally, the utility of the collected data was increased by linking the KNHANES data with related data from other government agencies or institutions and making the overall data publicly available on the official website of KNHANES (https://knhanes.kdca.go.kr). Additional long-term plans are being developed, including plans to continue producing nationwide health indicators and invigorating the utilization of the KNHANES data.

## INTRODUCTION

The Korea National Health and Nutrition Examination Survey (KNHANES) is an ongoing surveillance system that was initiated to produce nationwide statistics regarding the health status, health behaviors, and food and nutrient consumption of the Korean population. The first survey was conducted in 1998, based on Article 16 of the National Health Promotion Act. The survey data are utilized as evidence in the development and evaluation of health policies (like the National Health Plan [HP]) and are publicly available to researchers [1-3].

In this article, the 20-year accomplishments of the KNHANES are summarized, and a plan is presented to improve the health statistics to reflect changes in the social and survey environments.

## ACCOMPLISHMENTS

**Figure 1.**
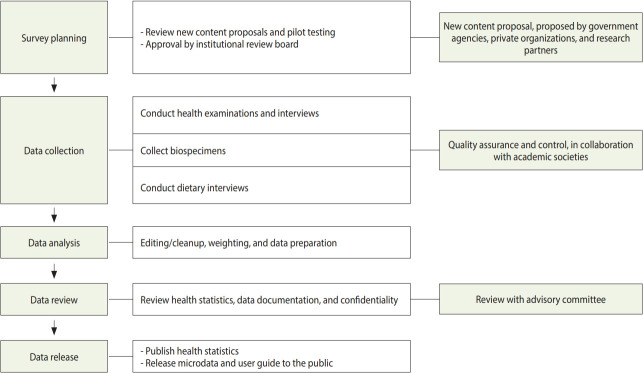
Operation of the Korea National Health and Nutrition Examination Survey.

Between 1998 and 2005, the survey was performed in a 3-year and 4-year cycle by the Korea Institute for Health and Social Affairs and the Korea Health Industry Development Institute. In 2005, the Korea Disease Control and Prevention Agency (KDCA, formerly Korea Centers for Disease Control and Prevention) participated in the health examination section of the survey. In 2006, the overall responsibility for the KNHANES was transferred to the KDCA and they have been conducting the survey since 2007 (Table 1 and Figure 1). Since the 1998 survey, data were collected from a cumulative total of 127,545 cases (response rate of 75.8%, based on health examination) (Table 2).

### Providing the evidence for health policy

The survey components were expanded in phases to reinforce the provision of information to guide health policies and programs (Table 3) (Supplementary Materials 1 and 2). A system for soliciting new content proposals for survey components was implemented on the KNHANES homepage. Demands for evidence on policy development were also identified through regular council meetings with relevant government agencies and institutions. These suggested proposals were assessed through internal and external reviews by the advisory committee, and components addressing the policy needs were introduced into the survey.

#### Expansion of the health examination survey

As the importance of evidence-based health and chronic disease policies has increased, the health examination components have been incrementally expanded since 2007 (Supplementary Material 1). In the surveys conducted between 1998 and 2005, the chronic disease statistics were limited to obesity, hypertension, diabetes, dyslipidemia, hepatitis B, pulmonary function (only in 2001), and heavy metals/cotinine (only in 2005) [4-7]. Beginning with the 2007 survey, the health examination components underwent phased expansions in collaboration with academic societies. In 2007, oral examinations were introduced for the first time, and pulmonary function testing was re-introduced. Tests for bone density, body fat, eye disease, and ear-nose-throat (ENT) diseases were introduced in 2008. An osteoarthritis test was introduced in 2009. Hence, the prevalence of oral diseases, chronic obstructive pulmonary disease, osteoporosis, eye diseases (glaucoma, diabetic retinopathy, etc.), ENT diseases (hearing loss, dizziness, etc.), and osteoarthritis [8-11] were estimated. Further, testing for blindness-causing eye diseases was re-introduced in 2017 and ENT diseases test focused on hearing ability, dizziness, and voice disorders was re-introduced in 2019.

Laboratory test components began to expand in 2010. Allergic disease tests was added in 2010, and tests for hepatitis C and thyroid disease were added in 2012 and 2013, respectively [12,13]. In 2014, tuberculin skin tests were performed to estimate the extent of tuberculosis infection. To assess the health risks associated with smoking, tobacco-specific nitrosamine was measured in 2016 (Supplementary Material 2).

Additionally, handgrip strength testing was included in 2014 to assess sarcopenia in the elderly population. Physical activity (PA) monitors were also introduced to objectively assess PA levels [14,15]. Recently, with the increasing need for evidence regarding the health impacts of climate change, levels of indoor air quality pollutants and exposure to hazardous environments were measured in 2020.

#### Expansion of the health interview survey

The health interview survey was enhanced by including the components needed to produce the HP indicators (Table 3) (Supplementary Material 1).

The EuroQol-5 dimension (EQ-5D) quality of life scale was introduced in 2005 to achieve the overarching goal of HP, that is, a healthy lifespan [16,17]. However, there was a high ceiling effect caused by participants, indicating that they did not have any problems in any of the question domains. Thus, the health-related quality of life instrument with eight items (HINT-8) was developed in 2014 as a quality of life scale reflective of Korean culture and was included in 2019 [18,19]. The alcohol use disorders identification test was introduced in 2005 to assess the prevalence of harmful alcohol use. To assess PA, the international PA questionnaire was introduced in 2005. With the increasing importance of assessing PA levels in different areas (work, leisure, and transportation), as well as assessing overall PA, the Korean version of the global PA questionnaire was included in 2014 [20]. Additionally, to accommodate the demand for sleep health, a sleep apnea screening tool was introduced in 2019 [21].

Following the 2015 changes in smoking cessation policy, the need to identify changes in smoking and smoking cessation behaviors arose. Accordingly, a panel consisting of smokers among KNHANES participants was created and annual follow-ups were performed from 2015 to 2020.

#### Expansion of the nutrition survey

The nutrition survey section was enhanced to facilitate research on nutritional factors associated with chronic diseases (Table 3) (Supplementary Material 1).

The food frequency questionnaire (FFQ) was transitioned to a semi-quantitative FFQ to collect long-term food and nutrient intake information, and a food security questionnaire was developed to enable in-depth analysis of nutritional equity. That is, to overcome the previous food-based FFQ (63 items) that was limited in its ability to assess the usual intake of food and nutrients, a dishbased, semi-quantitative FFQ (112 items) was introduced in 2012 [22,23]. To better assess the food security level, an 18-item scale was developed, based on the United States household food security survey module, and introduced in 2012 [24].

To facilitate research on the nutritional risk factors, nutritional biomarkers including vitamin D (2007) and vitamins A and E, and folic acid (2016) were added. In addition, a nutrient database that provides the nutrient values for dietary data reported in the KNHANES was developed, and intake of fatty acids (2015), cholesterol, fiber (2016), and sugar (2019) were newly produced [25- 27]. With the goal of expanding the database by adding one nutrient per year, plans were made to augment the database regarding folic acid (2020), vitamins D and E (2021), and others. Additionally, because more people are consuming dietary supplements, data have been collected regarding the details of dietary supplements since 2010.

### Improving the survey system

#### Establishment of the continuous annual survey system

The KDCA modified the survey system to produce annual health information to ensure the timeliness of the health statistics (Table 1). The 1st (1998) and 2nd (2001) phases of the KNHANES were conducted over a short period in a 3-year cycle. The 3rd phase was conducted in 2005, a year later than the original plan, as the survey contents were revised to produce the indicators required for the interim evaluation of the HP2010 goals. The 2001 survey results were the most recent data available prior to mid-2006, when the 2005 survey results were reported. To improve the timeliness of the statistics, beginning with the 2007 survey (the 4th phase), the survey system was changed to make it a continuous annual survey, enabling the production of annual statistics. A 3-year rolling sampling cycle was introduced to ensure timeliness by producing annual nationwide statistics. Simultaneously, this approach also enabled the production of indicators for which statistical power was limited by the small annual sample size; the new approach permitted the combination of 2-3 years of data. In the 2005 survey and those preceding it, the number of participants in the different sections of the KNHANES varied. This was improved by having every participant participate in all three sections of the survey, beginning in 2007. Accordingly, the sample size for the health examination section increased approximately 3-fold, leading to stability in the chronic disease statistics.

Next, the KDCA changed the survey period from a short-term to a continuous, annual (48 weeks) format to increase accuracy (including reducing any seasonal bias) in the statistics. During the period when short-term surveys were conducted, the field staff were also hired for similarly short terms. With the change to the continuous annual survey, teams of full-time field staff were assembled, and the data were collected by a total of 44 trained fulltime field staff.

#### Development of internal and external quality control

Public-private partnerships were formed with relevant academic societies to maintain expertise in quality assurance and control. Approximately 30 expert advisory committees composed of over 180 experts participated in quality assurance and control. Specifically, full-time field staff training, guideline development, and field quality control tasks were performed in collaboration with relevant academic societies. Collaboration with academic societies was formalized through memoranda of understanding and research contracts in quality control. The quality control details are publicly available on the KNHANES homepage (https://knhanes.kdca.go.kr). Advisory committee members are also responsible for reviewing survey components, methods, and results.

#### Improvement of the data collection method

Specially designed and equipped mobile examination centers (MEC) were introduced in 2008 when survey components requiring equipment were included, as described in the section “expansion of the health examination survey.” The use of MEC improved data quality because the data were collected using standardized equipment and environments. In addition, in 2013 and 2014, health interviews and nutrition surveys were changed from paper-based personal interviews to computer-assisted personal interviewing (CAPI), which increased the data accuracy by introducing process standardization and decreasing the response burden on the participants.

### Facilitating data utilization

#### Availability of KNHANES data

In December of the year following survey completion, a report (“*Health Statistics*”) was published, and raw data became publicly available through the KNHANES homepage. Additionally, the KDCA research data center is operated to support research conducted using variables that are not publicly available, such as survey dates and geographic areas (Table 4). Furthermore, beginning in 2011, annual data user workshops were held to support researchers using KNHANES data by providing training on data utilization. Consequently, 27,903 researchers have downloaded the KNHANES data and more than 3,800 articles have been published.

#### Establishment of the criteria and reference values

As the KNHANES data are representative of the Korean population, they are used to establish reference values and criteria. Using the data, reference ranges for bone density, body fat mass, and muscle strength, as well as normal predicted values for lung diffusion capacity have been developed for each sex and age at the national level [1,10,28]. Newly produced statistics regarding the intake of various nutrients, distribution of anthropometric data, and obesity prevalence in the pediatric population have been utilized in the development of ‘the Dietary Reference Intakes for Koreans’ and ‘the Korean National Growth Charts’, respectively [29,30].

#### Data linkage activity

A weakness of cross-sectional surveys is a challenge associated with identifying the risk factors of chronic disease and their temporal relationship with the disease. Since 2007, the participant’s informed consent has allowed data to be linked with data available at other government agencies or institutions. Presently, KNHANES data are linked with Statistics Korea (cause of death statistics) and the Korea National Cancer Center (cancer registry data). Of the 81,503 individuals who participated in the 2007-2016 surveys, 75,016 individuals were linked to the cause of death statistics and 74,977 individuals to the cancer registry data. The data linked to the cause of death statistics became publicly available in 2020, and the database is expected to be augmented with newly linked data every year (Table 4).

A linked database was also constructed with the air quality data provided by the Ministry of Environment. A validation study is currently underway on the linked data, and the linked database will be released. To activate research on health-related social and physical environments, data linkages will be expanded with health information (including geographic information and social attributes) of other government agencies or institutions.

#### Collection and sharing of human bioresources

Informed consent obtained from participants has also allowed the collection of bioresources to build biobanks (Table 5).

Based on the biospecimens collected to date, the rates of severe fever with thrombocytopenia syndrome, hepatitis A, hepatitis E, and genetic variants of alcohol-metabolizing enzymes have been reported. Presently, biochemical markers including homocysteine, and apolipoproteins have been measured using biospecimens between 2013 and 2015 KNHANES. After reviewing the specimen stability and quality control, the results will be released to the public. These biospecimens are also provided to researchers requesting human bioresources through services for the access and sharing desk of the National Biobank of Korea (http://nih.go.kr/biobank).

#### Participation in collaborative research

The KDCA joined the NCD Risk Factor Collaboration in 2015, and the Global Dietary Database Consortium in 2019 [31-37]. Every year, the KDCA shares the KNHANES raw data, food recipes, and other data with these collaborators, and is involved in studies on the trends and risk factors associated with non-communicable disease, using the shared data.

### Amendments to the National Health Promotion Act

The decrees and rules of the National Health Promotion Act related to the KNHANES have been amended. The amendments included those that permitted the current annual survey system, added dentists as field staff members, and allowed laboratory technicians and radiology technicians to work as field staff. Currently, work is underway to amend the name of survey from ‘the Korea National Nutrition Survey’ to ‘the Korea National Health and Nutrition Examination Survey’ in Article 16 of the National Health Promotion Act.

## FUTURE DIRECTIONS

**Figure 2.**
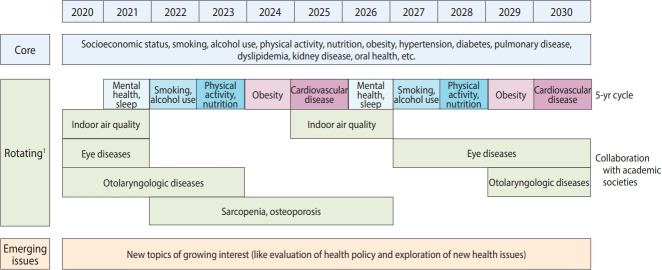
Survey components of the Korea National Health and Nutrition Examination Survey. ^1^ Rotating items may be changed by plan revision.

Multifaceted plans for the long-term development of the survey are being prepared to revise the survey system to fit the changing survey environment. The changes also aim to explore emerging public health issues, and to produce the health statistics needed to develop health policies in a timely manner (Figure 2) [38,39].

### Improving the survey system

The continuous annual survey system will be carefully revised after reviewing the comparability with the existing statistics and the feasibility. Reductions in sampling bias are expected by additional methods (such as home visits) to facilitate the participation of people with limited mobility due to chronic diseases or accidents. In the National Health and Nutrition Examination Survey [40] and the Health Survey for England [41], vulnerable groups are oversampled to predict health problems in these groups. If there is a need to address health issues in specific groups in Korea, oversampling or additional surveys linked to the main KNHANES will be explored over time.

A study on the need for follow-up surveys to overcome weaknesses in the current, cross-sectional survey and to identify the incidence rates and natural history of chronic disease suggested that it is necessary to establish a new independent survey system. Hence, for the time being, efforts are being made to expand the data sources linked with the KNHANES data, as described previously [42]. Additionally, because “smoker’s panel” was considered to be a cost-effective way to examine changes in smoking behavior and factors associated with the behavioral changes, additional KNHANES panels are planned, as necessary.

### Expanding the survey components

Currently, new survey components are developed by soliciting new content proposals via the KNHANES homepage and regular council meetings with relevant government agencies and institutions. However, to include more wide-ranging components, the demand for new components should be identified using more diverse methods. More active participation in the process is planned through regular notices by official letters on proposals to relevant academic societies and private sectors. Moreover, there are plans to expand council meetings to additional relevant institutions where the KNHANES data are utilized to monitor the targets for the objectives of HP and submit health indicators to WHO and Organization for Economic Cooperation and Development.

#### Survey cycle

Survey components are categorized as core (surveyed annually), rotating (surveyed once per cycle), and emerging topics (surveyed once due to a policy change or public health demand). However, the criteria for categorizing the components and determining the survey cycles remain unclear. A plan exists to establish a survey cycle for each component to avoid unnecessary repetition of certain components and to facilitate the inclusion of new components. First, components included in other surveys and those that can be analyzed by links to other available institutional data will be removed. Next, the survey cycle criteria will be determined for each component, and the existing components will be reclassified using these criteria. Further, as new components are added to the survey, their appropriate survey cycles will also be determined.

#### In-depth survey

Both the National Health Interview Survey [43] and the Health Survey for England [41] include in-depth surveys that are periodically chosen for their relevance to health-related issues. In our survey, examinations of eye and ENT diseases were performed through in-depth surveys. Currently, survey components on health equity, mental health, and health indicators of the elderly are being developed for future in-depth surveys. In addition, priority will be given to reviewing new indicators to evaluate the objectives added for the fifth Health Plan (HP2030) and areas to be added to the current survey (like social support systems, health-related environment factors, and factors related to pre-stage of chronic diseases). The criteria for introducing in-depth components or their survey cycles will be established, and the components, based on priority, will be added.

### Improving survey methods

#### Utilization of web-based, wearable devices

Questionnaires on socioeconomic status, medical condition, health care utilization in the health interview survey, and nutrition survey were administered using CAPI. Questionnaires on health behaviors were administered using computer-assisted self-interviewing (CASI) methodology. Survey methods other than CAPI and CASI should be explored considering the time burden on participants and the limited space within the MEC. In the case of young and middle-aged participants, it is also necessary to complete the questionnaires using computers or smartphones before visiting MEC. Presently, studies are being conducted to compare the response rates and results of major health indicators with those of the current system.

PA monitors were used between 2014 and 2017 to objectively assess PA levels over 7-day periods. In the future, wearable devices will be used to assess sleep hours and blood pressure as well as PA levels.

#### Nutrition surveys using mobile examination centers

The nutrition survey was performed through home visits to the households of participants in the health examination and health interview surveys. Recently, the need to revise the nutrition survey method has arisen due to increasing home visit refusals and privacy protection, and participant’s burden required to complete a separate nutrition survey. At present, the development of a MEC-based survey system for nutrition surveys is being researched. The nutrition survey method will be modified in consideration of results such as the response rate and comparability with the existing data.

### Increasing the use of data

The incremental expansion of the databases that link KNHANES data with data available from other government agencies or institutions, as well as the construction of biobanks and sharing of biospecimens, will continue, as described in the Accomplishments section.

#### Data accessibility

The changes in survey components and the reasons for the changes are described, to the extent possible, in a report (“*Health Statistics*”). However, the changes may not be clearly described due to the vast volume and frequency of the changes made to survey areas and components. Accordingly, plans are being made to construct a survey component management database, integrated survey data storage, and a data visualization. This will provide researchers to simplify data search and visualization.

Data user workshop has remained unchanged since 2011, except for 2016 when an additional workshop was organized to describe the dietary data analysis. Future workshops will be designed to cover diverse content according to the analysis level and survey areas. Currently, if researchers want to utilize non-public KNHANES data or data linked to other government agencies, they are required to conduct their analysis at the research data center. To enhance data accessibility, a remote analysis system is planned, that will enable researchers to analyze data remotely.

#### In-depth analysis

Presently, the KNHANES results are provided as a report (“*Health Statistics*”) where the current status and trends are presented by sex, age, and income level, limiting the use of the data for all but expert users. To address this problem, 20-year trends in health behaviors and chronic diseases were visualized in fact sheets in 2020, making the data easy to understand for the public. To further increase the utilization of various aspects of the data, multi-angle, in-depth data analysis is necessary. Collaboration with relevant academic societies is planned that will allow them to develop relevant indicators and conduct analyses that reflect user needs.

## CONCLUSION

Over the past 20 years, the KNHANES has achieved the timely production of nationwide statistics by establishing a continuous annual survey system, vigorous production of health indicators by expanding survey components, enhancement of statistical accuracy by improving survey methods, and improvement of data utilization by making raw data and linked databases publicly available. In the future, we plan to develop long-term plans that incorporate the advances discussed in this article to allow the continued production of health indicators and further support utilization of the data.

### Ethics statement

This study was approved by the Institutional Review Board of the KDCA (2007-2014, 2018). For certain year (2015-2017), ethical approval was waived by the Act (Article 2, Paragraph 1) and Enforcement Regulation (Article 2, Paragraph 2, item 1) of Bioethics and Safety Act.

## Figures and Tables

**Table 1.** Reorganization of the Korea National Health and Nutrition Examination Survey

Survey system	Year	Contents		Effects
		Before	After	
Survey cycle	2007	Conducted periodically (3-yr cycle) for 2-3 mo	Conducted continuous annual survey	Improve timeliness, reduce seasonal variation
Sample size	2007	10,000 Individuals/3-yr (based on health examination)	10,000 Individuals/1-yr (based on health examination)	Improve statistical power
Field site	2008	Available public place (depending on primary sampling unit)	Mobile examination center	Standardize survey environments and equipment
Field staff	2007	Short-term hired researcher	Full-time staff	Improve data quality
Survey operation	2007	Conducted by a private institution	Conducted by KDCA	Stabilize survey system, improve data quality

KDCA, Korea Disease Control and Prevention Agency.

**Table 2.** Data collection for the Korea National Health and Nutrition Examination Survey (KNHANES)

Survey	Participants, n			Response rate, %		
	Health interview	Health examination	Nutrition survey	Health interview	Health examination	Nutrition survey
KNHANES I (1998)	39,060	9,771	11,267	-	89.8	-
KNHANES II (2001)	37,769	9,770	10,051	-	77.3	81.0
KNHANES III (2005)	34,145	7,597	9,047	-	70.2	80.5
KNHANES IV (2007-2009)	23,632		22,137	74.5		81.8
KNHANES V (2010-2012)	24,173		22,949	76.5		82.3
KNHANES VI (2013-2015)	21,724		20,686	74.1		81.7
KNHANES VII (2016-2018)	23,162		21,287	73.1		80.8
KNHANES VIII (2019)	7,716		7,151	71.0		79.3

**Table 3.** Extension of survey components in the Korea National Health and Nutrition Examination Survey

Year of introduction	Components	Indicators
Health examination survey		
2001	Pulmonary function test	Chronic obstructive pulmonary disease prevalence
2005	Mercury, lead, cadmium, cotinine	Environmental exposure
2007	Dental examination	Dental caries, periodontal disease prevalence
	Insulin	Diabetes-related indicator
	Vitamin D	Vitamin D deficiency
	Arsenic, manganese	Environmental exposure
2008	Bone density, body composition	Bone density/body fat distributions, osteoporosis prevalence
	PTH, ALP	Osteoporosis-related indicators
	Ophthalmology examination	Glaucoma, cataracts, macular degeneration, diabetic retinopathy prevalence
	Otolaryngologic disease examination	Hearing loss, dizziness, voice disorder prevalence (2019)
2009	Arthritis body measures	Osteoarthritis prevalence
2010	Immunoglobulin E-allergens	Allergic disease prevalence
	Iron, ferritin (2007), TIBC	Iron-deficiency anemia prevalence
2011	Microalbumin	Chronic kidney disease prevalence
2012	Hepatitis C, hepatitis A (2015)	Hepatitis prevalence
2013	TSH, free T4, TPOAb, Iodine	Thyroid disease prevalence
2014	Grip strength	Muscle strength, sarcopenia prevalence
	TST	Tuberculosis infection rate
	Physical activity monitor	Physical activity
2016	NNAL	Harmful effects of smoking
	Uric acid	Gout prevalence
2020	PM_2.5_, formaldehyde, TVOCs, CO_2_	Indoor air quality
Health interview survey		
2005	EQ-5D, EQ-VAS	Quality of life
	AUDIT	Alcohol use disorder
	IPAQ	Physical activity
2014	GPAQ	Physical activity
	PHQ-9	Depression symptom
2015	Smokers’ panel	Monitoring of smoking cessation policy
2019	HINT-8	Quality of life
	STOP-Bang	Sleep apnea
Nutrition survey		
2010	Dietary supplements	Nutrient intake by dietary supplements
2012	Semiquantitative food frequency questionnaire	Dietary risk factors for chronic disease
	Household food security survey module	Food insecurity
2015	Nutrient database	Fatty acids (2015), cholesterol (2016), dietary fiber (2016), sugar (2019), folic acid (2020) intakes
2016	Vitamin A, E, folic acid (serum)	Nutritional biomarker distribution

PTH, parathyroid hormone; ALP, alkaline phosphatase; TIBC, total iron-binding capacity; TSH, thyroid-stimulating hormone; free T4, free thyroxine; TPOAb, thyroid peroxidase antibody; TST, tuberculin skin test; NNAL, 4-(methylnitrosamino)-1-(3-pyridyl)-1-butanol; PM, particulate matter; TVOCs, total volatile organic compounds; CO_2_, carbon dioxide; EQ-5D, EuroQol-5 dimension; EQ-VAS, EuroQol visual analogue scale; AUDIT, alcohol use disorders identification test; IPAQ, International Physical Activity Questionnaire; GPAQ, Global Physical Activity Questionnaire; PHQ-9, Patient Health Questionnaire-9; HINT-8, health-related quality of life instrument with 8 items; STOP-Bang, snoring, tired, observed, blood pressure, body mass index, age, neck circumference, gender.

**Table 4.** Data release and linkage of Korea National Health and Nutrition Examination Survey (KNHANES)

Released or linked data		Year	n	Description
Public use data release				
	KNHANES	1998-2018	208,705	1998-2018 KNHANES microdata
Data linkage				
	Linked causes of death statistics	2007-2016	75,016	Date, cause of death
	Linked cancer registration statistics	2007-2016	74,977	Year (registered in cancer registration program), age (diagnosed with cancer), cancer sites and others
	Linked air quality data	2007-2017	85,108	PM_2.5_, PM_10_, sulfur dioxide, nitrogen dioxide, ozone, carbon monoxide, temperature, humidity and others

PM, particulate matter.

**Table 5.** Biospecimens of Korea National Health and Nutrition Examination Survey (KNHANES)

Survey	Biospecimen, n		
	Serum	Plasma	DNA
KNHANES IV (2007-2009)	18,825	14,982	14,289
KNHANES V (2010-2012)	19,687	12,354	13,071
KNHANES VI (2013-2015)	9,365	8,949	8,789
KNHANES VII (2016-2018)	9,430	9,578	9,467
KNHANES VIII (2019)	4,400	4,403	4,389

## SUPPLEMENTARY MATERIALS

Supplementary Material 1. Examination components of the Korea National Health and Nutrition Examination Survey

Supplementary Material 2. Laboratory components of the Korea National Health and Nutrition Examination Survey

Korean version is available at http://www.e-epih.org/.
