# Supplementary material for: Korea National Health and Nutrition Examination Survey, 20th anniversary: accomplishments and future directions
Source: Epidemiol Health. 2021 Apr 19;43:e2021025. doi: 10.4178/epih.e2021025 (PMC8289475; doi:10.4178/epih.e2021025)
Supplement: Supplementary Material 1. — Examination components of the Korea National Health and Nutrition Examination Survey [file epih-43-e2021025-suppl1.docx]

**Supplementary Material 1. Examination components of the Korea National Health and Nutrition Examination Survey**

| **Survey** | | **Components** | **1998** | **2001** | **2005** | **2007** | **2008** | **2009** | **2010** | **2011** | **2012** | **2013** | **2014** | **2015** | **2016** | **2017** | **2018** | **2019** | **2020** |
| --- | --- | --- | --- | --- | --- | --- | --- | --- | --- | --- | --- | --- | --- | --- | --- | --- | --- | --- | --- |
| **Health**  **interview** | | Socioeconomic status |  |  |  |  |  |  |  |  |  |  |  |  |  |  |  |  |  |
|  |  | Smoking |  |  |  |  |  |  |  |  |  |  |  |  |  |  |  |  |  |
|  |  | Alcohol use |  |  |  |  |  |  |  |  |  |  |  |  |  |  |  |  |  |
|  |  | AUDIT |  |  |  |  |  |  |  |  |  |  |  |  |  |  |  |  |  |
|  |  | Physical activity |  |  |  |  |  |  |  |  |  |  |  |  |  |  |  |  |  |
|  |  | IPAQ |  |  |  |  |  |  |  |  |  |  |  |  |  |  |  |  |  |
|  |  | GPAQ |  |  |  |  |  |  |  |  |  |  |  |  |  |  |  |  |  |
|  |  | Mental health |  |  |  |  |  |  |  |  |  |  |  |  |  |  |  |  |  |
|  |  | PHQ-9 |  |  |  |  |  |  |  |  |  |  |  |  |  |  |  |  |  |
|  |  | Quality of life: EQ-5D |  |  |  |  |  |  |  |  |  |  |  |  |  |  |  |  |  |
|  |  | EQ-VAS |  |  |  |  |  |  |  |  |  |  |  |  |  |  |  |  |  |
|  |  | HINT-8 |  |  |  |  |  |  |  |  |  |  |  |  |  |  |  |  |  |
|  |  | Sleep: STOP-Bang |  |  |  |  |  |  |  |  |  |  |  |  |  |  |  |  |  |
|  |  | Chronic disease conditions |  |  |  |  |  |  |  |  |  |  |  |  |  |  |  |  |  |
|  |  | Health screening |  |  |  |  |  |  |  |  |  |  |  |  |  |  |  |  |  |
|  |  | Vaccination |  |  |  |  |  |  |  |  |  |  |  |  |  |  |  |  |  |
|  |  | Healthcare utilization |  |  |  |  |  |  |  |  |  |  |  |  |  |  |  |  |  |
|  |  | Activity limitations |  |  |  |  |  |  |  |  |  |  |  |  |  |  |  |  |  |
|  |  | ADL, IADL |  |  |  |  |  |  |  |  |  |  |  |  |  |  |  |  |  |
|  |  | Obesity and weight control |  |  |  |  |  |  |  |  |  |  |  |  |  |  |  |  |  |
|  |  | Oral health |  |  |  |  |  |  |  |  |  |  |  |  |  |  |  |  |  |
|  |  | Reproductive health |  |  |  |  |  |  |  |  |  |  |  |  |  |  |  |  |  |
|  |  | Safety |  |  |  |  |  |  |  |  |  |  |  |  |  |  |  |  |  |
|  |  | Family history of disease |  |  |  |  |  |  |  |  |  |  |  |  |  |  |  |  |  |
| **Nutrition**  **survey** | | 24-hour dietary recall |  |  |  |  |  |  |  |  |  |  |  |  |  |  |  |  |  |
|  |  | Food frequency questionnaire (FFQ) |  |  |  |  |  |  |  |  |  |  |  |  |  |  |  |  |  |
|  |  | Dish-based, semi-quantitative FFQ |  |  |  |  |  |  |  |  |  |  |  |  |  |  |  |  |  |
|  |  | Food security |  |  |  |  |  |  |  |  |  |  |  |  |  |  |  |  |  |
|  |  | HFSSM |  |  |  |  |  |  |  |  |  |  |  |  |  |  |  |  |  |
|  |  | Dietary behavior |  |  |  |  |  |  |  |  |  |  |  |  |  |  |  |  |  |
| **Survey** | **Components** | **1998** | **2001** | **2005** | **2007** | **2008** | **2009** | **2010** | **2011** | **2012** | **2013** | **2014** | **2015** | **2016** | **2017** | **2018** | **2019** | **2020** |  |
| **Health**  **Examination** | Body measurements |  |  |  |  |  |  |  |  |  |  |  |  |  |  |  |  |  |  |
|  | Blood pressure |  |  |  |  |  |  |  |  |  |  |  |  |  |  |  |  |  |  |
|  | Spirometry |  | * |  |  |  |  |  |  |  |  |  |  |  |  |  |  |  |  |
|  | Dental caries |  |  |  |  |  |  |  |  |  |  |  |  |  |  |  |  |  |  |
|  | Periodontal disease |  |  |  |  |  |  |  |  |  |  |  |  |  |  |  |  |  |  |
|  | DEXA: Bone density |  |  |  |  |  |  |  |  |  |  |  |  |  |  |  |  |  |  |
|  | Body composition |  |  |  |  |  |  |  |  |  |  |  |  |  |  |  |  |  |  |
|  | Chest X-rays |  |  |  |  |  |  |  |  |  |  |  |  |  |  |  |  |  |  |
|  | Knee, hip-joint X-rays |  |  |  |  |  |  |  |  |  |  |  |  |  |  |  |  |  |  |
|  | Tuberculin skin tests |  |  |  |  |  |  |  |  |  |  |  |  |  |  |  |  |  |  |
|  | Visual acuity, autorefraction |  |  |  |  |  |  |  |  |  |  |  |  |  |  |  |  |  |  |
|  | Retinal photos |  |  |  |  |  |  |  |  |  |  |  |  |  |  |  |  |  |  |
|  | Visual fields |  |  |  |  |  |  |  |  |  |  |  |  |  |  |  |  |  |  |
|  | Intraocular pressure |  |  |  |  |  |  |  |  |  |  |  |  |  |  |  |  |  |  |
|  | Intraocular lens master |  |  |  |  |  |  |  |  |  |  |  |  |  |  |  |  |  |  |
|  | Optical coherence tomography |  |  |  |  |  |  |  |  |  |  |  |  |  |  |  |  |  |  |
|  | Audiometry |  |  |  |  |  |  |  |  |  |  |  |  |  |  |  |  |  |  |
|  | Balance |  |  |  |  |  |  |  |  |  |  |  |  |  |  |  |  |  |  |
|  | Nasal endoscopy |  |  |  |  |  |  |  |  |  |  |  |  |  |  |  |  |  |  |
|  | Laryngoscopy |  |  |  |  |  |  |  |  |  |  |  |  |  |  |  |  |  |  |
|  | Voice disorder test |  |  |  |  |  |  |  |  |  |  |  |  |  |  |  |  |  |  |
|  | Grip strength test |  |  |  |  |  |  |  |  |  |  |  |  |  |  |  |  |  |  |
|  | Physical activity monitor |  |  |  |  |  |  |  |  |  |  |  |  |  |  |  |  |  |  |
|  | Indoor air quality |  |  |  |  |  |  |  |  |  |  |  |  |  |  |  |  |  |  |

AUDIT, alcohol use disorders identification test; IPAQ, international physical activity questionnaire; GPAQ, global physical activity questionnaire; PHQ-9, patient health questionnaire-9; EQ-5D, EuroQol-

5 dimension; EQ-VAS, EuroQol visual analogue scale; HINT-8, health-related quality of life instrument with 8 items: STOP-Bang: snoring, tired, observed, blood pressure, body mass index, age, neck

circumference, gender; ADL/IADL, activities of daily living/instrumental Activities of daily living; FFQ, food frequency questionnaire; HFSSM, household food security survey module; DEXA, dual

energy x-ray absorptiometry

*: Unleased to the public

**Supplementary Material 2. Laboratory components of the Korea National Health and Nutrition Examination Survey**

| **Components** | **1998** | **2001** | **2005** | **2007** | **2008** | **2009** | **2010** | **2011** | **2012** | **2013** | **2014** | **2015** | **2016** | **2017** | **2018** | **2019** | **2020** |
| --- | --- | --- | --- | --- | --- | --- | --- | --- | --- | --- | --- | --- | --- | --- | --- | --- | --- |
| Alanine aminotransferase (blood) | ○ | ○ | ○ | ○ | ○ | ○ | ○ | ○ | ○ | ○ | ○ | ○ | ○ | ○ | ○ | ○ | ○ |
| Alkaline phosphatase (blood) | - | - | - | - | ○ | ○ | ○ | ○ | - | - | - | - | - | - | - | - | - |
| Arsenic (urine) | - | - | - | ○* | ○ | ○ | - | - | - | - | - | - | - | - | - | - | - |
| Aspartate aminotransferase (blood) | ○ | ○ | ○ | ○ | ○ | ○ | ○ | ○ | ○ | ○ | ○ | ○ | ○ | ○ | ○ | ○ | ○ |
| C reactive protein (blood) | - | - | - | - | - | - | - | - | - | - | - | ○ | ○ | ○ | ○ | - | - |
| Cadmium (blood) | - | - | ○ | ○* | ○ | ○ | ○ | ○ | ○ | ○ | - | - | ○ | ○ | - | - | - |
| Cotinine (urine) | - | - | ○* | ○* | ○ | ○ | ○ | ○ | - | - | ○ | ○ | ○ | ○ | ○ | ○ | ○ |
| Creatinine (blood) | ○ | ○ | ○ | ○ | ○ | ○ | ○ | ○ | ○ | ○ | ○ | ○ | ○ | ○ | ○ | ○ | ○ |
| Creatinine (urine) | - | - | - | ○* | ○ | ○ | ○ | ○ | ○ | ○ | ○ | ○ | ○ | ○ | ○ | ○ | ○ |
| Ferritin (blood) | - | - | - | ○ | ○ | ○ | ○ | ○ | ○ | - | - | - | - | - | - | - | - |
| Folic acid (blood) | - | - | - | - | - | - | - | - | - | - | - | - | ○ | ○ | ○ | - | - |
| Formaldehyde (urine) | - | - | - | - | - | - | - | - | - | - | - | - | - | - | - | - | ○ |
| Free thyroxine (blood) | - | - | - | - | - | - | - | - | - | ○ | ○ | ○ | - | - | - | - | - |
| Gamma-glutamyl transferase (blood) | - | - | - | - | - | - | ○ | ○ | - | - | - | - | - | - | - | - | - |
| Glucose (blood) | ○ | ○ | ○ | ○ | ○ | ○ | ○ | ○ | ○ | ○ | ○ | ○ | ○ | ○ | ○ | ○ | ○ |
| Glycohemoglobin (blood) | ○ | ○ | ○ | ○ | ○ | ○ | ○ | ○ | ○ | ○ | ○ | ○ | ○ | ○ | ○ | ○ | ○ |
| HDL-cholesterol (blood) | ○ | ○ | ○ | ○ | ○ | ○ | ○ | ○ | ○ | ○ | ○ | ○ | ○ | ○ | ○ | ○ | ○ |
| Hematocrit (blood) | ○ | ○ | ○ | ○ | ○ | ○ | ○ | ○ | ○ | ○ | ○ | ○ | ○ | ○ | ○ | ○ | ○ |
| Hemoglobin (blood) | ○ | ○ | ○ | ○ | ○ | ○ | ○ | ○ | ○ | ○ | ○ | ○ | ○ | ○ | ○ | ○ | ○ |
| Hepatitis A antibody (blood) | - | - | - | - | - | - | - | - | - | - | - | ○ | - | - | - | - | - |
| Hepatitis B anti-HBs (blood) | ○ | - | - | - | - | - | - | - | - | - | - | - | - | - | - | - | - |
| Hepatitis B HBsAg (blood) | ○ | ○ | ○ | ○ | ○ | ○ | ○ | ○ | ○ | ○ | ○ | ○ | ○ | ○ | ○ | ○ | ○ |
| Hepatitis C antibody (blood) | - | - | - | - | - | - | - | - | ○ | ○ | ○ | ○ | ○ | ○ | ○ | ○ | ○ |
| Hepatitis C RNA (blood) | - | - | - | - | - | - | - | - | ○ | ○ | ○ | ○ | - | - | - | - | - |
| Immunoglobulin E-allergens (blood) | - | - | - | - | - | - | ○ | - | - | - | - | - | - | - | - | ○ |  |
| Insulin (blood) | - | - | - | ○ | ○ | ○ | ○ | - | - | - | - | ○ | - | - | - | ○ | ○ |
| Iodine (urine) | - | - | - | - | - | - | - | - | - | ○ | ○ | ○ | - | - | - | - | - |
| Iron (blood) | - | - | - | - | - | - | ○ | ○ | ○ | - | - | - | - | - | - | - | - |
| Lead (blood) | - | - | ○ | ○* | ○ | ○ | ○ | ○ | ○ | ○ | - | - | ○ | ○ | - | - | - |
| Manganese (blood) | - | - | - | ○* | ○ | ○ | - | - | - | - | - | - | - | - | - | - | - |
| Mercury (blood) | - | - | ○ | ○* | ○ | ○ | ○ | ○ | ○ | ○ | - | - | ○ | ○ | - | - | - |
| Microalbumin (urine) | - | - | - | - | - | - | - | ○ | ○ | ○ | ○ | - | - | - | - | ○ | ○ |
| Nickle (blood) | - | - | - | - | - | - | - | - | - | - | - | - | - | ○ | - | - | - |

| **Components** | **1998** | **2001** | **2005** | **2007** | **2008** | **2009** | **2010** | **2011** | **2012** | **2013** | **2014** | **2015** | **2016** | **2017** | **2018** | **2019** | **2020** |
| --- | --- | --- | --- | --- | --- | --- | --- | --- | --- | --- | --- | --- | --- | --- | --- | --- | --- |
| NNAL (urine) | - | - | - | - | - | - | - | - | - | - | - | - | ○ | ○ | ○ | - | - |
| Parathyroid hormone (blood) | - | - | - | - | ○ | ○ | ○ | ○ | - | - | - | - | - | - | - | - | - |
| Potassium (urine) | - | - | - | - | - | - | - | - | - | - | - | - | ○ | ○ | ○ | ○ | ○ |
| Sodium (urine) | - | - | - | - | ○ | ○ | ○ | ○ | - | - | ○ | ○ | ○ | ○ | ○ | ○ | ○ |
| Thyroid peroxidase antibody (blood) | - | - | - | - | - | - | - | - | - | ○ | ○ | ○ | - | - | - | - | - |
| Thyroid-stimulating hormone (blood) | - | - | - | - | - | - | - | - | - | ○ | ○ | ○ | - | - | - | - | - |
| Total cholesterol (blood) | ○ | ○ | ○ | ○ | ○ | ○ | ○ | ○ | ○ | ○ | ○ | ○ | ○ | ○ | ○ | ○ | ○ |
| Total iron binding capacity (blood) | - | - | - | - | - | - | ○ | ○ | ○ | - | - | - | - | - | - | - | - |
| Triglycerides (blood) | ○ | ○ | ○ | ○ | ○ | ○ | ○ | ○ | ○ | ○ | ○ | ○ | ○ | ○ | ○ | ○ | ○ |
| Uric acid (blood) | - | - | - | - | - | - | - | - | - | - | - | - | ○ | ○ | ○ | ○ | ○ |
| Vitamin A (Retinol, blood) | - | - | - | - | - | - | - | - | - | - | - | - | ○ | ○ | ○ | - | - |
| Vitamin D (25-hydroxyvitamin D, blood) | - | - | - | ○* | ○ | ○ | ○ | ○ | ○ | ○ | ○ | - | - | - | - | - | - |
| Vitamin E (α-tocopherol, blood) | - | - | - | - | - | - | - | - | - | - | - | - | ○ | ○ | ○ | - | - |
| Volatile organic compounds (VOC) (urine) | - | - | - | - | - | - | - | - | - | - | - | - | - | - | - | - | ○ |
| Zinc (blood) | - | - | - | - | - | - | ○ | - | - | - | - | - | - | - | - | - | - |

HDL, high-density lipoprotein; LDL, low-density lipoprotein; HBsAg, hepatitis B surface antigen; anti-HBs, antibodies against hepatitis B surface antigens; NNAL, 4-(methylnitrosamino)-1-(3-pyridyl)-1-butanol

*: Unleased to the public
