# Supplementary material for: Korea National Health and Nutrition Examination Survey, 20th anniversary: accomplishments and future directions
Source: Epidemiol Health. 2021 Apr 19;43:e2021025. doi: 10.4178/epih.e2021025 (PMC8289475; doi:10.4178/epih.e2021025)
Supplement: Supplementary file 2 [file epih-43-e2021025-suppl2.docx]

**Title:**

**(국문) 국민건강영양조사 20년(1998-2018): 성과 및 발전방안**

**(영문) Korea National Health and Nutrition Examination Survey, 20^th^ Anniversary: Accomplishments and future directions**

**Running title**: Twenty-year accomplishments in KNHANES

**Authors:**

오경원^1^, 김윤정^1^, 권상희^1^, 김소연^1^, 윤성하^1^, 박수연^1^, 이연경^2^, 김영택^3^, 박옥^1^, 정은경^4^

Kyungwon Oh^1^, Yoonjung Kim^1^, Sanghui Kweon^1^, Soyeon Kim ^1^, Sungha Yun^1^, Suyeon Park^1^, Yeon-Kyeng Lee^2^, Youngtaek Kim^3^, Ok Park^1^, and Eun Kyeong Jeong^4^

**Affiliations:**

^1^질병관리청 만성질환관리국 건강영양조사분석과, ^2^질병관리청 의료안전예방국 의료감염 관리과, ^3^충남대학교 병원, ^4^질병관리청

^1^Division of Health and Nutrition Survey and Analysis, Bureau of Chronic Disease Prevention and Control, Korea Disease Control and Prevention Agency, Cheongju, Korea

^2^Division of Healthcare Association Infection Control, Bureau of Healthcare Safety and Immunization, Korea Disease Control and Prevention Agency, Cheongju, Korea

^3^Public Health Medical Service Office, Chungnam National University Hospital, Daejeon, Korea

^4^Korea Disease Control and Prevention Agency, Cheongju, Korea

**Correspondence:** Eun Kyeong Jeong

Korea Disease Control and Prevention Agency, 187 Osongsaengmyeong 2-ro, Heungduk-gu, Cheongju 28159, Korea

E-mail: jeongek@korea.kr

**초록**

국민건강영양조사는 국가의 건강정책 수립 및 평가의 근거 생산을 위해 1998년에 시작한 이후 현재까지 질병관리청 주관으로 실시하고 있다. 지난 20년동안 전문조사수행팀에 의한 연중 조사 체계 구축, 조사항목 단계적 확대, 관련 학회와 공동조사 및 질 관리, 자문단 운영 등 조사체계를 개편하여 시의성, 정확성을 갖춘 국가단위 건강통계 및 건강정책 수립에 필요한 근거 생산을 강화하였다. 또한 국민건강영양조사 홈페이지(<http://knhanes.kdca.go.kr)>를 통해 수집자료 및 타 기관과 연계자료를 공개하여 활용도를 향상하였다. 향후 국가 단위의 건강지표를 지속적으로 추가 생산하고 조사자료 및 결과활용을 활성화하는 방향 등을 포함한 중장기 계획을 수립, 추진할 계획이다.

**주제어**: 국민건강영양조사, 건강행태, 영양, 만성질환

**개요**

국민건강영양조사는 국민건강증진법제16조에 근거하여 1998년부터 우리 국민의 건강수준, 건강행태, 식품 및 영양소 섭취 실태에 대한 국가단위 통계를 산출하는 조사사업이다. 조사 자료와 통계는 국민건강증진종합계획(National Health Plan, HP)의 목표 설정 및 평가, 건강증진 프로그램 개발 등 건강정책의 근거자료, 보건분야의 연구자료로 활용되고 있다 [1, 2, 3]. 본 논문에서는 국민건강 영양조사 20년간의 성과를 요약하고, 고령화, 1인가구의 증가 등 사회환경, 조사여건 변화를 반영한 건강통계 생산의 발전 방안을 논의하고자 한다.

**성과**

국민건강영양조사는 2005년 조사까지 3∼4년 주기로 한국보건사회연구원과 한국보건 산업진흥원에 의해 수행되었다. 질병관리청은 2005년 조사의 일부인 검진조사 수행기관으로 참여하였으며 2006년에 조사 전체를 이관받아 2007년 조사부터 현재까지 동일한 수행체계로 운영하고 있다(Table 1, Figure 1). 1998년 조사 이후 127,545건(검진조사 기준, 참여율 75.8%)의 자료를 수집하였다(Table 2).

**1. 건강정책 근거 생산 강화**

국민건강영양조사 목적인 건강정책 수립 및 평가의 근거 산출 기능을 강화하기 위해 조사항목을 단계적으로 확대하였다(Table 3, Supplementary Materials 1 and 2). 홈페이지를 통해 조사항목에 관한 수요접수 체계를 구축하고 접수된 수요에 관한 내부 검토, 분과 및 조정 자문위원회를 거쳐 도입 여부를 결정하고 있다. 또한 정책부서와 정례적으로 협의체를 운영하여 정책 수요를 파악, 도입하고 있다.

**1) 검진조사 확대**

근거 기반의 건강 및 만성질환 정책 개발의 중요성이 강조됨에 따라 검진 기반의 만성질환 통계 생산에 중점을 두고 2007년부터 단계적으로 검진항목을 확대하였다(Supplementary Material 1).

해당 연도에만 실시한 폐기능검사(2001년), 중금속 및 코티닌검사(2005년)를 제외하고, 1998년∼2005년 조사는 비만, 고혈압, 당뇨병, 이상지질혈증, B형간염에 관한 통계만을 산출하였다 [4∼7]. 2007년부터 만성질환에 관한 지표 추가를 목표로 학회와 협력하여 단계적으로 조사항목을 확대하였다. 2007년 구강검사 도입, 폐기능검사 재개, 2008년 골밀도 및 체지방검사, 안질환검사, 이비인후질환검사, 2009년 골관절염검사를 도입하여 구강질환, 만성폐쇄성폐질환, 골다공증, 안질환 (녹내장, 당뇨망막병증, 나이관련황반변성 등), 이비인후질환(난청, 어지럼증 등), 골관절염 유병에 관한 국가 단위의 통계를 생산하였다 [8∼11]. 2017년에 대한안과학회와 실명 유발과 관련된 안질환조사를, 2019년에 대한이비인후과학회와 청력, 어지럼증, 음성장애조사 중심의 이비인후질환 조사를 재개하여 수행 중에 있다.

2010년 이후에는 진단의학검사 항목을 확대하여 2010년 알레르기질환, 2012년 C형간염, 2013년 갑상선질환 유병률을 산출하였다 [12, 13]. 2014년 투베르쿨린피부반응검사(Tuberculin Skin Test)를 실시하여 결핵감염률을 산출하였고, 흡연에 의해 유발된 건강위해 평가를 위해 2016년에 NNAL((4-(methylnitrosamino)-1-(3-pyridyl)-1-butanol))을 도입하였다(Supplementary Material 2).

또한, 고령화 사회에 대비하여 노인 인구의 신체기능을 평가할 수 있는 악력검사를 도입(2014년)하여 근력, 근감소증 현황을 모니터링하고 있고, 설문도구의 단점(기억에 의한 오류, 낮은 민감도 등)을 보완하고 객관적 방법에 의한 신체활동 수준 평가를 위해 가속도계(Actigraph, 2014년)를 도입하였다 [14, 15]. 최근 미세먼지, 기후변화 등으로 인한 건강영향에 관한 근거 자료의 필요성이 대두됨에 따라 2020년 하반기부터 가정의 초미세먼지, 포름알데히드, 총휘발성화합물 측정과 생체지표(포름알데히드, 총휘발성화합물) 분석을 통해 가정 실내의 공기 질과 유해환경 노출 수준을 모니터링하고 있다.

**2) 건강설문조사 확대**

건강설문은 국민건강증진종합계획 지표 산출에 필요한 항목을 확대하는 방향으로 개선하였으며 항목 추가 시 타당도가 검증된 조사도구를 도입하여 지표의 정확도, 국가간 비교 가능성을 향상하였다(Table 3, Supplementary Material 1).

국민건강증진종합계획의 총괄 목표인 건강수명과 관련하여 2005년에 삶의 질 측정도구인 EQ-5D(EuroQol-5 Dimension)를 도입하였다 [16, 17]. 그러나 모든 영역에서 문제가 없다고 응답하는 천장효과가 높게 나타나 건강상태 구분의 적절성, 문화적 차이의 고려 필요성이 제기되었다. 2014년에 우리나라 문화를 반영한 삶의 질 도구(Health-related quality of life instrument with 8 items, HINT-8)를 개발, 2016년 가치 평가 연구를 완료한 후 2019년부터 추가로 도입하여 격년으로 삶의 질을 평가하고 있다 [18, 19]. 음주 부문은 음주폐해 수준을 파악하기 위해 AUDIT(Alcohol Use Disorders Identification Test)을 2005년에 도입하였다. 신체활동의 경우도 2005년에 신체활동설문 (International Physical Activity Questionnaire, IPAQ)을 도입하여 근거 기반의 신체활동 지침 개발에 활용하였다. 이후 신체활동 총량뿐만 아니라 영역별로 구분한 신체활동 수준 평가도 중요하게 됨에 따라 2013년에 활동 영역별(일, 여가, 이동) 중등도 및 고강도 신체활동을 평가하는 신체활동설문(Global Physical Activity Questionnaire, GPAQ) 한글판을 개발하여 2014년에 도입하였다 [20]. 또한, 수면건강 조사 수요를 반영하여 폐쇄성수면무호흡증 선별 도구의 유용성 평가를 실시한 후 2019년에 수면무호흡평가설문(Snoring, Tired, Observed, Pressure, Body mass index, age, neck, gender, STOP-Bang questionnaire)을 도입하였다[21]. 2015년 금연정책 변화(담뱃값 인상 등)에 따른 흡연 및 금연행태 변화 파악의 필요성이 제기되어 국민건강영양조사 참여자 중 흡연자를 대상으로 패널을 구축, 매년 1회 추적조사(2015년∼2020년)를 실시하였다.

**3) 영양조사 확대**

영양조사는 검진 및 건강설문조사 자료와 연계 분석, 만성질환과 관련된 영양요인 연구 활성화 등 자료 활용을 확대하는 방향으로 개선하였다(Table 3, Supplementary Material 1).

식품섭취빈도조사는 장기간의 식품 및 영양소 섭취수준 파악이 가능하도록 반정량 식품섭취빈도조사로 전환하고, 식품안정성조사도 영양부문의 형평성 심층분석이 가능하도록 조사도구를 개발하였다. 즉, 기존 단순식품섭취빈도조사(63개 항목)의 식품 및 영양소 섭취량 평가 시 제한점을 보완하기 위해 음식 기반의 반정량식품섭취빈도조사지(112개 항목)를 개발하고 2011년 사전조사 결과(소요시간, 대상자 조사부담 등)를 반영하여 2012년에 19~64세 대상으로 도입하였다 [22, 23]. 식품안정성조사는 2005년에 1개 항목으로 도입하였으나 안정성 확보 정도를 추가로 평가하기 위해 미국의 식품안정성 조사도구(Household Food Security Survey Module, HFSSM)를 기초로 18개 항목의 측정 도구를 개발하여 2012년에 도입하였다 [24].

심뇌혈관질환, 암 등 만성질환과 관련된 영양요인 연구를 활성화하기 위해 2007년 비타민 D, 2016년 비타민 A, E, 엽산 등 영양관련 생체지표를 도입하였다. 국민건강영양조사 기반의 영양 성분 DB를 신규로 구축하여 2015년 지방산, 2016년 콜레스테롤과 식이섬유, 2019년 당 섭취수준에 관한 통계를 생산하였다 [25∼27]. 매년 한 개씩 영양성분 DB 확대를 목표로 2020년 엽산, 2021년 이후에는 비타민 D, E 등에 관한 DB를 구축할 계획이다. 또한 식이보충제 복용이 증가함에 따라 2010년부터 식이보충제 섭취내용을 조사하여 보충제로 인한 영양소 섭취 수준을 평가하고 있다.

**2. 조사체계 및 방법 개선**

**1) 연중조사 체계 구축**

질병관리청은 조사를 이관 받은 후 우선 통계의 시의성을 확보하기 위해 매년 조사를 수행하는 체계로 개편하였다(Table 1). 제1기, 제2기 조사는 당시 국민건강증진법에 근거하여 3년 주기로 각각 1998년, 2001년에 단기간(2∼3개월) 실시하였다. 제3기 조사는 국민건강증진종합 계획(HP2010)에서 기 설정한 목표의 중간평가에 필요한 지표 생산에 적절하도록 조사내용 재검토와 수정을 위해 계획한 2004년보다 일정이 늦춰진 2005년에 실시하였다. 2005년 조사결과가 발표된 2006년 상반기까지 2001년 조사결과가 가용한 최신 자료인 점으로 인한 통계의 시의성 부족을 개선하기 위해 2007년(제4기) 조사부터 매년 조사수행 및 통계생산 체계로 변경하였다. 표본 설계는 3년 단위의 순환표본을 도입하여 매년 국가단위 통계를 생산하여 시의성을 확보함과 동시에 표본 수로 인해 통계산출에 제한이 있는 지표는 2∼3년 자료를 통합하여 산출이 가능하도록 하였다. 2005년 조사까지는 조사부문별(건강설문, 영양, 검진조사)로 대상자 수의 차이가 있었으나 2007년 조사부터 대상자가 모든 조사에 참여하도록 개선하였다. 이로 인해 검진조사의 표본 수가 약 3배정도 확대되어 만성질환 통계의 안정성이 확보되었고 조사 부문별 연계 분석을 활성화하는 기반이 마련되었다.

다음으로는 통계의 계절적인 편향 보완 등 정확성 향상을 위해 단기간의 조사(제1∼2기 11∼12월, 제3기 4∼6월)를 연중(48주 조사) 실시로 변경하였다. 단기간의 조사로 인해 2005년 조사까지는 단기채용 조사원이 조사를 수행하였으나 연중조사 체계로 개편하면서 전문조사 수행팀을 구성하여 일정 기간의 교육 프로그램(조사 개요, 조사 부문별 조사지침, 현장실습 등)을 이수한 조사원에 의한 자료수집으로 통계의 질을 제고, 유지하고 있다. 전문조사수행팀은 4팀으로 구성하였고, 각 팀에 혈압측정, 신체계측, 채혈, 폐기능검사, 구강검사 담당 각 1인, 건강설문조사 담당 2인, 영양조사 담당 2인을 포함하였으며, 이후 안질환, 이비인후질환조사 등 항목 확대에 따라 현재 4팀, 총 44명이 조사를 수행하고 있다.

**2) 조사 질관리 강화**

조사 부문별 전문성 확보를 위해 관련학회와 질관리, 자문단(30여개 분과, 180여명) 운영 등 민·관 협력체계를 구축하였다. 특히 전문조사수행팀 교육, 지침서 개발, 현장수행 질관리 등을 관련학회(대한결핵및호흡기학회, 대한고혈압학회, 대한예방치과·구강보건학회, 대한안과학회, 대한 이비인후과학회, 대한진단검사의학회 등)와 협력하여 공동으로 실시하고 있다. 업무협약(MOU) 체결, 정책연구용역을 통해서 학회와 협력하고 있으며(협력학회는 조사항목에 따라 변동), 질관리에 관한 자세한 사항은 국민건강영양조사 홈페이지(https:// knhanes.kdca.go.kr)에 공개하여 자료 활용 시 참고할 수 있도록 하고 있다. 자문단은 또한 조사항목, 방법 및 조사결과 검토에도 참여하고 있다.

**3) 조사방법 개선**

‘검진조사 확대’에서 기술한 바와 같이 2008년에 검진 장비가 필요한 조사항목이 포함됨에 따라 검진차량을 도입하였고, 표준화된 장비와 조사환경에서의 자료 수집으로 인해 조사의 질이 향상되는 효과가 있었다. 검진조사 항목의 주기적인 변경에 따른 장비 장착, 혈압 측정(소음, 진동수준 조절), 설문조사 환경(응답의 비밀 보장을 위한 독립적이고 폐쇄적 공간) 등 조사환경 개선을 위해 2013년과 2019년에 조사차량을 신규로 도입하였다. 또한, 2013년과 2014년에 기존의 종이를 이용한 건강설문 및 영양조사를 컴퓨터를 이용한 조사방법(Computer Assisted Personal Interviewing, CAPI)으로 변경하였고 이는 조사과정 표준화에 의한 정확성 향상과 함께 대상자의 응답 부담을 감소하는 효과가 있었다.

**3. 자료활용 활성화**

**1) 원시자료 공개**

조사 완료한 다음 해 12월에 통계집을 발간하고 국민건강영양조사 홈페이지를 통해 원시자료를 공개하고 있다. 조사일, 조사지역 등 비공개변수를 활용한 연구 지원을 위해 질병관리청 내 학술연구자료처리실을 추가로 운영하고 있다(Table 4). 또한 2011년부터 매년 조사항목, 표본설계, 자료분석 시 고려사항을 주요 내용으로 자료활용 워크숍을 개최(총 11회, 4,180명 참석)하여 연구자의 조사자료 활용을 지원하고 있다. 그 결과 27,903명의 연구자가 조사자료를 활용하여 약 3,800편 이상의 논문이 발표되고 있다.

**2) 기준 및 참고치 마련**

국민건강영양조사가 우리 국민을 대표하는 자료라는 장점으로 인해 참고치 마련 또는 기준 설정 시 활용하고 있다. 골밀도검사, 근력검사, 폐기능검사 결과를 기초로 국가 단위의 성별, 연령별 골밀도, 체지방, 근력 참고치와 폐확산능 정상 예측치 등을 발표하였다 [1, 10, 28]. 신규로 산출한 지방산, 콜레스테롤, 식이섬유, 당 등 영양소 섭취 수준 자료는 ‘한국인의 영양소 섭취 기준’제정, 소아청소년 신체계측 자료의 분포와 비만 유병률 자료는 ‘소아청소년 성장도표’ 제정 시 활용하였다 [29, 30].

**3) 타 기관 자료와 연계DB 구축**

만성질환과 관련된 위험요인과 선후관계 규명이 어려운 단면조사의 단점을 보완하기 위하여 2007년부터 조사 대상자로부터 타 기관(통계청, 국립암센터, 국민건강보험공단, 건강보험 심사평가원) 연계에 관한 동의를 구득하고 있다. 이 기관들 중 현재까지는 통계청(사망원인통계), 국립암센터(중앙암등록자료)와 연계DB를 구축하였다. 사망원인통계의 경우 2007~2016년 조사에 참여한 81,503명 중 75,016명에 대한 자료(연계율 92.0%), 중앙암등록자료의 74,977명의 자료(연계율 91.9%)를 연계하였다. 사망원인통계와 연계자료는 시범연구, 개인정보 영향평가 등을 거친 후 2020년 상반기에 공개하였으며 매년 연계자료를 추가하여 구축할 계획이다(Table 4).

기관에서 공개하고 있는 자료 중에서는 환경부 대기환경정보([www.airkorea.or.kr)](http://www.airkorea.or.kr)fh)에 CMAQ(Community Multiscale Air Quality) 방법을 적용하여 생산한 유해환경자료(미세먼지, 초미세먼지, 이산화질소, 이산화황 등)와 연계DB를 구축하였다. 구축한 연계자료의 타당성 검토가 현재 진행 중이며 검토 완료 후 공개할 예정이다. 이와 유사한 방법으로 타 기관의 공개자료와 연계를 단계적으로 확대할 계획이다. 즉, 건강과 관련된 사회·물리적 환경에 관한 연구 활성화를 고려하여 통계청, 기상청, 교통부 등 관련 기관이 보유하고 있는 정보(인구, 기후, 지리정보 등)와 연계도 기획·추진할 예정이다.

**4) 인체자원 구축 및 분양**

질병관리청으로 조사를 이관한 2007년부터 대상자의 동의를 취득하여 대상자의 혈액으로부터 인체자원을 수집하여 구축하고 있다(’07~’18년, 혈청 56,592건, 혈장 45,697건, DNA 43,980건) (Table 5).

구축한 인체자원을 통해 중증열성혈소판감소증후군 항체 양성률, A형 및 E형 간염 항체 양성률, 알코올 대사관련 효소의 유전자 변이 분포 등 자료의 장점을 활용한 결과를 발표하고 있고, 국민건강영양조사 진단의학검사의 정확도 향상을 위한 분석 등에 활용하고 있다. 현재 2013∼2015년 국민건강영양조사 검체에 대한 호모시스테인, Apolipoprotein A, B, Apoprotein(a) 등에 관한 분석을 추가로 실시 중이며 검체 안정성, 질관리 및 분석 결과를 검토 후 향후 공개할 예정이다. 또한, 연구자가 국립중앙인체자원은행의 인체자원분양데스크(http://nih.go.kr/biobank)를 통해 인체자원 분양신청 시 분양위원회 심의를 거쳐 제공하고 있다.

**5) 국제 공동연구 참여**

2015년에 만성질환 추이, 위험요인, 질병부담에 관한 연구인 NCD Risk Factor Collaboration, 2019년에 영양관련 만성질환 현황 추이와 질병부담에 관한 연구인 Global Dietary Database Consortium에 각각 참여하고 있다 [31∼37]. 매년 국민건강영양조사 원시자료, 음식 레시피 등 수집한 자료를 공동연구팀과 공유하고 비만, 당뇨병, 고혈압, 심뇌혈관질환 유병 추이 및 위험요인에 관한 연구에 참여하고 있다.

**4. 국민건강증진법 개정**

국민건강영양조사 관련된 국민건강증진법 시행령과 시행규칙을 개정하였다. 조사 주기를 3년에서 매년으로 조정하고 조사원에 치과의사를 추가하였고, 의사, 간호사, 영양사, 치과의사 이외에 임상병리사, 방사선사 등이 조사원으로 참여할 수 있는 근거를 포함하는 등 현 조사체계에 적합하도록 개정하였다. 현재 국민건강증진법제16조의‘국민영양조사’를 ‘국민건강영양조사’ 로 개정을 추진하고 있다.

**발전방안**

국민건강영양조사는 1998년 첫 조사를 시작한 이후 국가 수준의 건강관련 통계를 지속적으로 산출하고 있다. 1인가구 증가, 개인정보 보호에 대한 민감성 증가 등 조사 여건의 변화로 인한 조사체계의 지속성과 노령화, 미세먼지 등 유해물질 노출, 식품산업 등 건강문제 변화를 유발할 수 있는 환경을 고려하여 새로운 건강문제 예측하고 관련 건강정책 개발에 필요한 시의성을 갖춘 통계 생산을 위해 중장기 발전 방안을 다각도로 기획하고 있다(Figure 2) [38, 39].

**1. 조사체계 개선**

2007년에 적용한 연중조사 체계를 기본으로 하되, 위에서 언급한 여러 환경의 변화를 고려하여 조사체계를 단계적으로 개선하고자 한다. 조사체계는 기존에 발표한 통계와의 비교 가능성과 실행 가능성(예산, 전문조사수행팀 운영 등)에 관한 검토 후 신중하게 개편할 계획이다.

대표성 유지 측면에서는, 인구 초고령화(2019년 기준, 65세이상 21.9%)로 인해 만성질환, 사고 등으로 인한 거동불능으로 이동검진차량 방문이 어려운 대상자를 고려한 참여 방안(별도 가구방문 등)을 마련하여 표본 편향을 방지할 계획이다. 미국의 국민건강영양조사(National Health and Nutrition Examination Survey, NHANES) [40], 영국의 건강조사(Health Survey for England, HSE) [41]는 건강취약집단(어린이, 노인, 소수 인종 등)의 건강문제 예측을 위해 과대표본추출방법을 이용하여 해당 통계를 산출하고 있다. 자료의 정책적 활용 측면에서 청소년, 노인, 다문화가정 등 특정 집단의 건강문제 발굴이 필요할 경우 위에 기술한 국외 사례를 참고하여 과대표본추출 또는 본 조사와 연계한 추가조사를 실시하는 방안을 장기적으로 검토할 계획이다.

현 단면조사의 단점을 보완하고 만성질환 발생 규모, 질환의 자연사 규명을 위한 추적조사 도입 방안을 검토한 결과 현 조사수행 체계와 유사한 규모의 조사체계 구축 필요성이 제안되어 [42],‘타 기관 자료와 연계DB 구축’에서 기술한 바와 같이 연계 자료원을 확대하는 방향으로 우선 추진하고 있다. 흡연자패널이 비용효과적으로 금연정책 변화에 따른 흡연행태, 변화와 관련된 요인을 규명할 수 있는 점을 고려하여, 필요 시 국민건강영양조사 기반의 추가 패널을 기획하여 단기 또는 장기로 운영할 계획이다.

**2. 조사항목 확대**

현재 국민건강영양조사 홈페이지, 정책부서와의 정례 협의를 통해 신규 조사항목을 개발하고 있지만 보다 광범위한 항목 도입을 위해 다양한 방법에 의한 수요 파악이 필요하다. 관련 학회의 적극적 참여 유도를 위해 학회를 대상으로 정기적으로 홍보를 실시하고, 국민건강증진 종합계획의 성과 평가, WHO, OECD 등 국제간 비교 자료로 활용됨을 고려하여 유관기관으로 정책협의체를 확대하여 운영할 계획이다.

**1) 조사주기**

현 조사에서 조사항목을 필수(지속적인 모니터링을 위해 매년 조사), 순환(주기적 조사), 기획(정책 변화, 보건분야 요구에 의한 일회성 조사)으로 분류하여 운영하고 있으나 항목을 구분하는 기준과 주기가 명확하게 수립되어 있지 않은 상황이다. 조사항목별 순환조사를 정착하여 불필요한 반복 조사를 방지하고 신규항목의 도입이 용이하도록 운영할 계획이다. 첫 단계로 타 조사에서 수행(예: 한국의료패널조사의 의료이용, 표본손상조사의 손상), 타 기관 자료와 연계 시 분석 가능한 항목(예: 국민건강보험공단, 건강보험심사평가원의 이환, 의료이용)은 축소하고자 한다. 다음으로 조사항목별 주기 설정을 위한 기준을 마련하고 기준에 따라 기존 항목은 재 분류, 신규 항목은 도입단계에서 주기를 설정하여 조사 시 적용하고자 한다.

**2) 심층조사**

미국 건강면접조사(National Health Interview Survey, NHIS) [43], 영국 건강조사 [41]의 경우 주기적으로 건강관련 이슈를 고려하여 심층영역을 선정, 조사를 실시하고 있다. 우리 조사도 안질환조사, 이비인후질환조사 등이 심층조사의 형식으로 수행하고 있다. 정책과의 연계 강화를 위해 심층조사 중장기 계획을 수립할 예정이며 향후 심층조사에 대비하여 정신건강, 건강형평성, 노인건강 영역에 관한 조사항목을 현재 개발 중에 있다. 또한, 제5차 국민건강증진종합계획 (HP2030)에 신규로 추가된 목표지표, 현 조사영역에서 보완이 필요하다고 제안된 사회지지체계, 건강관련 환경요인, 만성질환 전 단계 관련요인 등을 조사항목으로 검토 중에 있으며, 심층조사 도입 및 주기 등에 관한 기준을 설정한 후 우선 순위가 따라 조사에 도입할 계획이다.

**3. 조사방법 개선**

**1) 웹기반, 웨어러블 기기 활용**

현재 건강설문조사 중 교육 및 경제활동, 이환 등의 설문과 영양조사는 CAPI로 실시되며, 흡연, 음주 등 건강행태 설문은 CASI(Computer Assisted Self-Interviewing)로 실시되고 있다. 대상자의 참여시간에 대한 부담, 협소한 검진차량 공간 등을 고려하여 CAPI, CASI 이외의 조사 방안에 대한 검토가 필요하다. 특히, 20~50대 조사대상의 경우 이동검진차량 방문 전에 컴퓨터나 스마트폰을 이용하여 조사에 참여하는 개선안을 고려할 수 있다. 현재 설문조사 방법에 따른 조사 참여율, 건강지표 결과 등을 비교하는 연구가 수행 중이며 이 연구결과를 반영하여 조사방법을 개선할 계획이다.

2014년에 신체활동량 측정을 위한 가속도계를 도입하여 7일 동안의 객관적 측정에 의한 신체활동 수준을 평가하였으나 낮은 참여율, 높은 조사중간 포기율, 기기 분실 및 고장과 같은 운영상의 어려움으로 인해 2017년까지만 조사하였다. 향후 웨어러블 기기를 활용한 방법을 보완하여 신체활동 및 좌식활동 수준, 수면시간을 평가할 계획이며, 혈압 측정, 식품섭취조사 등으로 확대하는 방안을 검토, 추진할 예정이다.

**2) 이동검진차량 기반의 영양조사**

영양조사는 검진 및 건강설문 조사를 완료한 대상자의 가구를 직접 방문하여 면접조사하는 방법으로 운영 중이다. 최근 개인정보 보호에 대한 인식 강화로 인한 대상자의 가구방문 거부, 검진조사 후 별도의 영양조사 추가 실시로 인한 대상자 부담, 전문조사수행팀의 안전 우려 등으로 인해 수행체계 개선의 필요성이 대두되었다. 현재 차량 기반의 영양조사 수행체계 개발에 관한 연구가 수행 중이며, 이 연구결과(참여율, 기존 결과와 비교 가능성 등)를 고려하여 조사방법 변경 여부를 결정할 계획이다.

**4. 자료활용 확대**

타 기관 자료와 연계DB, 인체자원 구축 및 분양은 ‘성과’에서 기술한 바와 같이 단계적으로 확대, 활성화 할 계획이다.

**1) 자료 접근성**

통계집에 가능한 한 조사항목 변경 여부 및 사유를 기술하고 있으나 방대한 양, 잦은 조사영역 및 항목 변화로 인해 연구자가 이를 명확하게 파악하기 어려울 수 있다. 이를 고려하여 조사항목별 조사년도, 지표 정의, 변경사항 등을 포함한 조사항목 관리 데이터베이스를 구축하고 조사자료 통합 저장, 지도기반 데이터 등 시각화서비스를 개발하여 연구자가 자료를 쉽게 검색하고 시각화된 결과를 얻을 수 있는 서비스를 제공할 계획이다.

연구자의 자료활용 편의를 위해 2011년 이후 자료활용 워크숍을 개최하고 있으나 내부 인력의 제한으로 인해 2016년(영양자료 분석을 위한 자료활용 워크숍 추가 개최)을 제외하고서는 매년 프로그램의 변화없이 동일하게 구성·운영 중이다. 이를 개선하여 분석 수준별, 조사 부문별로 다양한 내용으로 구성한 워크숍을 기획·개최할 계획이다. 또한 연구자가 국민건강영양 조사 비공개자료(조사 날짜 및 지역 등), 통계청 등 타 기관 자료와 연계자료를 활용하고자 할 시 질병관리청 내 학술연구자료처리실에서 분석하게 되어 있어 접근성이 낮은 제한점이 있다. 이를 개선하기 위해 원격분석시스템(연구자가 자료분석 후 결과만 반출)을 구축 및 운영할 예정이다.

**2) 심층분석**

국민건강영양조사 결과는 보고서(통계집)의 형태로 제공되고 있는데 성별, 연령별, 소득수준별 현황 및 추이 제시에 그쳐 전문가 집단을 제외한 활용도는 제한적이다. 이를 고려하여 20년간의 건강행태 및 만성질환 추이를 요약하여 팩트시트(fact sheet) 형식으로 시각화하여 2020년에 발간하였다. 그러나 주요한 활용처인 국민건강증진종합계획 및 건강증진사업 성과 평가 이외의 다양한 측면의 활용을 촉진하기 위해 조사자료에 대한 다각적인 심층 분석이 필요하다. 이는 내부 인력으로만 추진하기에 제한적이어서 관련 학회와 협력하여 국민, 정책활용자 등 이용자 요구를 반영한 분석 기획, 지표 발굴 후 분석 결과를 발간, 홍보할 계획이다.

**결론**

지난 20년동안 국민건강영양조사는 연중조사체계 구축을 통한 시의성을 갖춘 국가단위 통계 생산, 단계적 조사항목 확대에 의한 건강행태 및 만성질환 정책 수립에 필요한 근거 생산 강화, 조사방법 개선에 의한 통계의 정확도 향상, 원시자료 및 타 기관 자료와 연계DB 공개를 통한 활용도 향상 등의 성과를 달성하였다. 향후 본 고에 기술한 내용을 포함한 중장기 계획을 수립하여 조사목적에 부합하는 지표를 지속적으로 생산하고 자료 활용을 활성화하는 방향으로 추진할 계획이다.

**윤리성명**

본 연구는 질병관리본부 연구윤리심의위원회 승인(2007-2014년, 2018년)과 생명윤리법 제2조제1호 및 동 시행규칙 제2조제2항제1호에 의해 심의 면제 받음(2015-2017년)

**CONFLICT OF INTEREST**

모든 저자는 본 연구에 대해 표명할 이해상충이 없음

**FUNDING**

없음

**ACKNOWLEDGEMENTS**

지난 20년동안 국민건강영양조사에 참여해주신 대상자께 감사드립니다. 또한, 조사를 전담하여 수행한 조사수행팀, 조사 지원과 자문을 주신 관련 학회 및 자문단에게도 감사드립니다.

**AUTHOR CONTRIBUTIONS**

Conceptualization: KO and EKJ. Data curation: YK, SK, SK, and SY. Formal analysis: SP. Funding acquisition: None. Project administration: YKL, YK, and OP. Writing-original draft: KO, YK, SK, SK, SY, SP. Writing-review & editing: KO, YKL, OP, and EKJ.

**ORCID**

Kyungwon Oh: [*http://orcid.org/0000-0001-8097-6078*](http://orcid.org/0000-0001-8097-6078); Yoonjung Kim: [*http://orcid.org/0000-0002-8418-0074*](http://orcid.org/0000-0002-8418-0074)*;* Sanghui Kweon: [*http://orcid.org/0000-0002-2678-8858*](http://orcid.org/0000-0002-2678-8858); Soyeon Kim: [*http://orcid.org/*](http://orcid.org/)*0000-0002-5027-1808;* Sungha Yun: *http://orcid.org/0000-0002-3624-4512*; Suyeon Park: *http://orcid. org/0000-0001-8134-8436*; Yeon-Kyeng Lee: *http://orcid.org/0000-0002-0828-455X*; Youngtaek Kim: [*http://orcid.org/0000-0003-0139-7620*](http://orcid.org/0000-0003-0139-7620); Ok Park: [*http://orcid.org/0000-0002-9477-9523*](http://orcid.org/0000-0002-9477-9523); Eun Kyeong Jeong: [*http://orcid.org/0000-0002-4386-201X*](http://orcid.org/0000-0002-9477-9523)

**REFERENCES**

1. Korea Centers for Disease Control & Prevention. Korea health statistics 2018: Korea National Health and Nutrition Examination Survey (KNHANES VII-3). 2019, p. 3-28 (Korean).

2. Kweon S, Kim Y, Jang MJ, Kim Y, Kim K, Choi S, et al. Data resource profile: The Korea National Health and Nutrition Examination Survey (KNHANES). Int J Epidemiol 2014;43:69-77.

3. Ministry of Health and Welfare of Korea. The fourth National Health Plan, 2016-2020. 2015, p. 33-41 (Korean).

4. Kim HJ, Kim Y, Cho Y, Jun BY, Oh KW. Trends in the prevalence of major cardiovascular disease risk factors among Korean adults: results from the Korea National Health and Nutrition Examination Survey, 1998-2012. Int J Cardiol 2014:174;64-72.

5. Jeon JY, Ko SH, Kwon HS, Kim NH, Kim JH, Kim CS, et al. Prevalence of diabetes and prediabetes according to fasting plasma glucose and HbA1c. Diabetes Metab J 2013;37:349-357.

6. Hwang YI, Yoo KH, Sheen SS, Park JH, Kim SH, Yoon HI, et al. Prevalence of chronic obstructive pulmonary disease in Korea: the result of Forth Korea National Health and Nutrition Examination Survey. Tuberc Respir Dis 2011;71(5):322-327.

7. Yoo KH, Kim YS, Sheen SS, Park JH, Hwang YI, Kim SH, et al. Prevalence of chronic obstructive pulmonary disease in Korea: the fourth Korea National Health and Nutrition Examination Survey, 2008. Respirology 2011;16:659-665.

8. Yoon KC, Mun GH, Kim SD, Kim SH, Kim CY, Park KH, et al. Prevalence of eye diseases in South Korea: data from the Korea National Health and Nutrition Examination Survey 2008-2009. Korean J Ophthalmol 2011;25:421-433.

9. Park KH, Lee SH, Koo JW, Park HY, Lee KY, Choi YS, et al. Prevalence and associated factors of tinnitus: data from the Korea National Health and Nutrition Examination Survey 2009-2011. J Epidemiol 2014;24:417-426.

10. Hong S, Oh HJ, Choi H, Kim JG, Lim SK, Kim EK, et al. Characteristics of body fat, body fat percentage and other body composition for Koreans from KNHANES IV. J Korean Med Sci 2011;26:1599-1605.

11. Park EJ, Joo IW, Jang MJ, Kim YT, Oh K, Oh HJ. Prevalence of osteoporosis in the Korean population based on Korea National Health and Nutrition Examination Survey (KNHANES), 2008-2011. Yonsei Med J 2014:55;1049-1057.

12. Lee H, Lee H, Cho Y, Oh K, Ki M. Changes in seroprevalence of hepatitis B surface antigen and epidemiologic characteristics in the Republic of Korea, 1998-2013. Epidemiol Health 2015;37:e2015055.

13. Kim WG, Kim WB, Woo G, Kim H, Cho Y, Kim TY, et al. Thyroid stimulating hormone reference range and prevalence of thyroid dysfunction in the Korean population: Korea National Health and Nutrition Examination Survey 2013 to 2015. Endocrinol Metab (Seoul) 2017;32:106-114.

14. Lee H, Lee M, Choi JY, Oh KW, Kim YJ, Kim SY. KNHANES actigraph raw data processing. The Korean Journal of Measurement & Evaluation in Physical Education and Sport Science 2018;20:83-94 (Korean).

15. Lee MY, Lee H, Choi JY, Oh KW, Kim YJ, Kim SY. Reliability evidences of accelerometer data to estimate physical activity participations: Korea National Health and Nutrition Examination Survey (KNHANES) in 2014. The Korean Journal of Measurement and Evaluation in Physical Education and Sport Science 2018;20:1-12 (Korean).

16. Lee YK, Nam HS, Chuang LH, Kim KY, Yang HK, Kwon IS, et al. South Korean time trade-off values for EQ-5D health states: modeling with observed values for 101 health states. Value Health. 2009;12(8):1187-93.

17. Korea Centers for Disease Control and Prevention. Validity and reliability evaluation for EQ-5D in Korea. 2011 , p. 37-65 (Korean).

18. Korea Centers for Disease Control and Prevention. Development of measurement tool for health-related quality of life in the Korea National Health and Nutrition Examination Survey. 2014, p. 38-97 (Korean).

19. Korea Centers for Disease Control and Prevention. Valuation of Korean health-related quality of life instrument with 8 items (HINT-8). 2017, p. 59-92 (Korean).

20. Korea Centers for Disease Control and Prevention. Development of the Korean version of global physical activity questionnaire and assessment of reliability and validity. 2013, p. 32-49 (Korean).

21. Korea Centers for Disease Control and Prevention. The usefulness of three screening questionnaires for obstructive sleep apnea in the Korea National Health and Nutrition Examination Survey. 2016, p. 18-29 (Korean).

22. Yun SH, Shim JS, Kweon SH, Oh, KW. Development of a food frequency questionnaire for the Korea National Health and Nutrition Examination Survey: data from the Fourth Korea National Health and Nutrition Examination Survey (KNHANES IV). J Nutr Health 2013;46:186-196 (Korean).

23. Kim DW, Song S, Lee JE, Oh K, Shim J, Kweon S, et al. Reproducibility and validity of an FFQ developed for the Korea National Health and Nutrition Examination Survey (KNHANES). Public Health Nutr 2014;18:1369-1377.

24. Kim HJ, Oh K. Household food insecurity and dietary intake in Korea: results from the 2012 Korea National Health and Nutrition Examination Survey. Public Health Nutr 2015;18:3317-3325.

25. Baek Y, Hwang JY, Kim K, Moon HK, Kweon S, Yang J, et al. Dietary intake of fats and fatty acids in the Korean population: Korea National Health and Nutrition Examination Survey, 2013. Nutr Res Pract 2015;9:650-657.

26. Park M, Kweon SH, Oh KW. Dietary Cholesterol intake in the Korea National Health and Nutrition Examination Survey (KNHANES) VI (2013-2015). Korean J Community Nutr 2017;22:520-528 (Korean).

27. Yeon SY, Oh KW, Kweon SH, Hyun TS. Development of a dietary fiber composition table and intakes of dietary fiber in Korea National Health and Nutrition Examination Survey (KNHANES). Korean J Community Nutr 2016;21(3):293~300 (Korean).

28. Kim K, Yun SH, Jang MJ, Oh KW. Body fat percentile curves for Korean children and adolescents: a data from the Korea National Health and Nutrition Examination Survey 2009-2010. J Korean Med Sci 2013;28:443-449.

29. Ministry of Health and Welfare. Dietary reference intakes for Koreans 2015. 2015, p. 2-19 (Korean).

30. Kim JH, Yun S, Hwang SS, Shim JO, Chae HW, Lee YJ, et al. The 2017 Korean national growth charts for children and adolescents: development, improvement, and prospects. Korean J Pediatr 2018;61:135-149.

31. Hajifathalian K, Ueda P, Lu Y, Woodward M, Ahmadvand A, Aguilar-Salinas CA, et al. A novel risk score to predict cardiovascular disease risk in national populations (Globorisk): a pooled analysis of prospective cohorts and health examination surveys. Lancet Diabetes Endocrinol 2015;3:339-355.

32. NCD Risk Factor Collaboration. Worldwide trends in body-mass index, underweight, overweight, and obesity from 1975 to 2016: a pooled analysis of 2,416 population-based measurement studies in 128·9 million children, adolescents, and adults. Lancet 2017;390:2627-2642.

33. Ueda P, Woodward M, Lu Y, Hajifathalian K, Al-Wotayan R, Aguilar-Salinas CA, et al. Laboratory-based and office-based risk scores and charts to predict 10-year risk of cardiovascular disease in 182 countries: a pooled analysis of prospective cohorts and health surveys. Lancet Diabetes Endocrinol 2017;5:196-213.

34. NCD Risk Factor Collaboration. Contributions of mean and shape of blood pressure distribution to worldwide trends and variations in raised blood pressure: a pooled analysis of 1018 population-based measurement studies with 88.6 million participants. Int J Epidemiol 2018;47:872-883i.

35. NCD Risk Factor Collaboration. Long-term and recent trends in hypertension awareness, treatment, and control in 12 high-income countries: an analysis of 123 nationally representative surveys. Lancet 2019;394:639-651.

36. NCD Risk Factor Collaboration. National trends in total cholesterol obscure heterogeneous changes in HDL and non-HDL cholesterol and total-to-HDL cholesterol ratio: a pooled analysis of 458 population-based studies in Asian and Western countries. Int J Epidemiol 2020;49:173-192.

37. NCD Risk Factor Collaboration. Rising rural body-mass index is the main driver of the global obesity epidemic in adults. Nature 2019;569:260-264.

38. Korea Centers for Disease Control and Prevention. A study for developing the national health survey system in Korea. 2006, p. 133-277 (Korean).

39. Korea Centers for Disease Control and Prevention. A study for improving the Korea National Health and Nutrition Examination Survey. 2018, p. 56-78 (Korean).

40. Centers for Disease Control and Prevention. National Health and Nutrition Examination Survey; 2020 [cited 2020 September 21]. Available from https://wwwn.cdc.gov/nchs/nhanes/analyticguidelines.aspx#sample-design.

41. NHS. Digital. Health Survey for England; 2020 [cited 2020 September 21]. Available from <https://digital.nhs.uk/data-and-information/publications/statistical/health-survey-for-england>.

42. Korea Centers for Disease Control and Prevention. Design and feasibility of the longitudinal study on the Korea National Health and Nutrition Examination Survey. 2008, p. 58-72 (Korean).

43. Centers for Disease Control and Prevention. National Health Interview Survey; 2020 [cited 2020 September 21]. Available from <https://www.cdc.gov/nchs/nhis/data-questionnaires-documentation.htm>.

**Table 1. Reorganization of the Korea National Health and Nutrition Examination Survey**

| Survey  system | Year | Contents | | Effects |
| --- | --- | --- | --- | --- |
|  |  | Before | After |  |
| Survey  cycle | 2007 | Conducted periodically (3-year cycle) for 2–3 months | Conducted continuous annual survey | Improve timeliness,  Reduce seasonal variation |
| Sample size | 2007 | 10,000 individuals/3-year (based on health examination) | 10,000 individuals/1-year (based on health examination) | Improve statistical power |
| Field site | 2008 | Available public place  (depending on primary sampling unit) | Mobile examination center | Standardize survey environments and equipment |
| Field staff | 2007 | Short-term hired researcher | Full-time staff | Improve data quality |
| Survey  operation | 2007 | Conducted by private institution | Conducted by KDCA | Stabilize survey system, Improve data quality |

KDCA, Korea Disease Control and Prevention Agency

**Table 2. Data collection for the Korea National Health and Nutrition Examination Survey**

| Survey | Participants (Number) | | | Response rate (%) | | |
| --- | --- | --- | --- | --- | --- | --- |
|  | Health  interview | Health  examination | Nutrition  survey | Health  interview | Health  examination | Nutrition  survey |
| KNHANES Ⅰ (1998) | 39,060 | 9,771 | 11,267 | - | 89.8 | - |
| KNHANES Ⅱ (2001) | 37,769 | 9,770 | 10,051 | - | 77.3 | 81.0 |
| KNHANES Ⅲ (2005) | 34,145 | 7,597 | 9,047 | - | 70.2 | 80.5 |
| KNHANES Ⅳ (2007–2009) | 23,632 | | 22,137 | 74.5 | | 81.8 |
| KNHANES Ⅴ (2010–2012) | 24,173 | | 22,949 | 76.5 | | 82.3 |
| KNHANES Ⅵ (2013–2015) | 21,724 | | 20,686 | 74.1 | | 81.7 |
| KNHANES Ⅶ (2016–2018) | 23,162 | | 21,287 | 73.1 | | 80.8 |
| KNHANES Ⅷ (2019) | 7,716 | | 7,151 | 71.0 | | 79.3 |

**Table 3. Extension of survey components in the Korea National Health and Nutrition Examination Survey**

| Year of Introduction | Components | Indicators | |
| --- | --- | --- | --- |
| Health examination survey | | | |
| 2001 | Pulmonary function test | | Chronic obstructive pulmonary disease prevalence |
| 2005 | Mercury, Lead, Cadmium, Cotinine | | Environmental exposure |
| 2007 | Dental examination | | Dental caries, Periodontal disease prevalence |
|  | Insulin | | Diabetes-related indicator |
|  | Vitamin D | | Vitamin D deficiency |
|  | Arsenic, Manganese | | Environmental exposure |
| 2008 | Bone density, Body composition | | Bone density/body fat distributions,  Osteoporosis prevalence |
|  | PTH, ALP | | Osteoporosis-related indicators |
|  | Ophthalmology examination | | Glaucoma, cataracts, macular degeneration,  diabetic retinopathy prevalence |
|  | Otolaryngologic disease examination | | Hearing loss, dizziness,  voice disorder prevalence (2019) |
| 2009 | Arthritis body measures | | Osteoarthritis prevalence |
| 2010 | Immunoglobulin E-allergens | | Allergic disease prevalence |
|  | Iron, Ferritin(2007), TIBC | | Iron-deficiency anemia prevalence |
| 2011 | Microalbumin | | Chronic kidney disease prevalence |
| 2012 | Hepatitis C, Hepatitis A(2015) | | Hepatitis prevalence |
| 2013 | TSH, Free T4, TPOAb, Iodine | | Thyroid disease prevalence |
| 2014 | Grip strength | | Muscle strength, sarcopenia prevalence |
|  | TST | | Tuberculosis infection rate |
|  | Physical activity monitor | | Physical activity |
| 2016 | NNAL | | Harmful effects of smoking |
|  | Uric acid | | Gout prevalence |
| 2020 | PM_2.5,_ Formaldehyde, TVOCs, CO_2_ | | Indoor air quality |
| Health interview survey | | | |
| 2005 | EQ-5D, EQ-VAS | | Quality of life |
|  | AUDIT | | Alcohol use disorder |
|  | IPAQ | | Physical activity |
| 2014 | GPAQ | | Physical activity |
|  | PHQ-9 | | Depression symptom |
| 2015 | Smokers’ panel | | Monitoring of smoking cessation policy |
| 2019 | HINT-8 | | Quality of life |
|  | STOP-Bang | | Sleep apnea |
| Nutrition survey | | | |
| 2010 | Dietary supplements | | Nutrient intake by dietary supplements |
| 2012 | Semi-quantitative food frequency questionnaire | | Dietary risk factors for chronic disease |
|  | Household food security survey module | | Food insecurity |
| 2015 | Nutrient database | | Fatty acids (2015), cholesterol (2016), dietary fiber (2016), sugar (2019), folic acid (2020) intakes |
| 2016 | Vitamin A, E, Folic acid(serum) | | Nutritional biomarker distribution |

PTH, parathyroid hormone; ALP, alkaline phosphatase; TIBC, total iron-binding capacity; TSH, thyroid-stimulating hormone; free T4, free thyroxine; TPOAb, thyroid peroxidase antibody; TST, tuberculin skin test; NNAL, 4-(methylnitrosamino)-1-(3-pyridyl)-1-butanol; PM, particulate matter; TVOCs, total volatile organic compounds; CO_2,_ carbon dioxide; EQ-5D, EuroQol-5 dimension; EQ-VAS, EuroQol visual analogue scale; AUDIT, alcohol use disorders identification test; IPAQ, international physical activity questionnaire; GPAQ, global physical activity questionnaire; PHQ-9, patient health questionnaire-9; HINT-8, health-related quality of life instrument with 8 items; STOP-Bang, snoring, tired, observed, blood pressure, body mass index, age, neck circumference, gender

**Table 4. Data release and linkage of Korea National Health and Nutrition Examination Survey**

| Released or linked data | Year | Number | Description |
| --- | --- | --- | --- |
| Public use data release |  |  |  |
| KNHANES | 1998–2018 | 208,705 | 1998-2018 KNHANES microdata |
| Data linkage |  |  |  |
| Linked causes of death statistics | 2007–2016 | 75,016 | Date, cause of death |
| Linked cancer registration  statistics | 2007–2016 | 74,977 | Year(registered in cancer registration program), age (diagnosed with cancer), cancer sites, etc. |
| Linked air quality data | 2007–2017 | 85,108 | PM_2.5,_ PM_10_, sulfur dioxide, nitrogen dioxide, ozone, carbon monoxide, temperature, humidity, etc. |

PM, particulate matter

**Table 5. Biospecimens of Korea National Health and Nutrition Examination Survey**

| Survey | Biospecimen (Number) | | |
| --- | --- | --- | --- |
|  | Serum | Plasma | DNA |
| KNHANES Ⅳ (2007–2009) | 18,825 | 14,982 | 14,289 |
| KNHANES Ⅴ (2010–2012) | 19,687 | 12,354 | 13,071 |
| KNHANES Ⅵ (2013–2015) | 9,365 | 8,949 | 8,789 |
| KNHANES Ⅶ (2016–2018) | 9,430 | 9,578 | 9,467 |
| KNHANES Ⅷ (2019) | 4,400 | 4,403 | 4,389 |

**Figure 1. Operation of the Korea National Health and Nutrition Examination Survey**

| **Survey Planning** |  |  | · Review new content proposals and pilot testing  · Approval by institutional review board |  | |  |  | | |
| --- | --- | --- | --- | --- | --- | --- | --- | --- | --- |
|  |  |  |  |  | |  | New content proposal,  proposed by government agencies, private  organizations, and research partners | | |
|  |  |  |  |  | |  |  |  |  |
|  |  |  |  |  | |  |  | | |
| **↓** |  | |  |  | |  | | | |
| **Data Collection** |  |  | · Conduct health examinations and interviews |  | |  |  | | |
|  |  |  |  |  | |  |  |  |  |
|  |  |  | · Collect biospecimens |  | |  | Quality assurance and control,  in collaboration with academic societies | | |
|  |  |  |  |  | |  |  |  |  |
|  |  |  | · Conduct dietary interviews |  | |  |  | | |
|  |  |  |  |  | |  |  |  |  |
| **↓** |  | |  |  | |  | | | |
| **Data Analysis** |  |  | · Editing/cleanup, weighting, and data preparation |  | |  | | | |
|  |  |  |  |  | |  | | | |
| **↓** |  | |  |  | |  |  | | |
| **Data Review** |  |  | · Review health statistics, data documentation, and confidentiality |  | |  | Review with advisory committee | | |
|  |  |  |  |  | |  |  |  |  |
| **↓** |  | |  |  | |  |  | | |
| **Data Release** |  |  | · Publish health statistics  · Release microdata and user guide to the public |  | |  | | |  |
|  |  |  |  |  |  | | |  | |

**Figure 2. Survey components of the Korea National Health and Nutrition Examination Survey**

|  |  | **2020** | **2021** | **2022** | **2023** | **2024** | **2025** | **2026** | **2027** | **2028** | **2029** | **2030** |  |
| --- | --- | --- | --- | --- | --- | --- | --- | --- | --- | --- | --- | --- | --- |
|  |  |  |  |  |  |  |  |  |  |  |  |  |  |
| **Core** |  | Socioeconomic status, smoking, alcohol use, physical activity, nutrition, obesity, hypertension, diabetes, pulmonary disease,  dyslipidemia, kidney disease, oral health, etc. | | | | | | | | | | |  |
|  |  |  |  |  |  |  |  |  |  |  |  |  |  |
| **Rotating** |  |  | Mental health, Sleep | Smoking,  alcohol use | Physical activity,  nutrition | Obesity | Cardiovascular disease | Mental health, Sleep | Smoking,  alcohol use | Physical activity,  nutrition | Obesity | Cardiovascular disease | 5-year  cycle |
|  |  | Indoor air quality | |  |  |  | Indoor air quality | |  |  |  |  |  |
|  |  | Eye diseases | |  |  |  |  |  | Eye diseases | | | | Collaboration with academic societies |
|  |  | Otolaryngologic diseases | | | |  |  |  |  |  | Otolaryngologic diseases | |  |
|  |  |  |  | Sarcopenia, Osteoporosis | | | | |  |  |  |  |  |
|  |  |  |  |  |  |  |  |  |  |  |  |  |  |
| **Emerging issues** |  | New topics of growing interest (like evaluation of health policy and exploration of new health issues) | | | | | | | | | | |  |

Rotating items may be changed by plan revision.
